# Identification and Characterization of a Stage Specific Membrane Protein Involved in Flagellar Attachment in *Trypanosoma brucei*

**DOI:** 10.1371/journal.pone.0052846

**Published:** 2013-01-15

**Authors:** Katherine Woods, Noirin Nic a’Bhaird, Clodagh Dooley, David Perez-Morga, Derek P. Nolan

**Affiliations:** 1 Molecular Parasitology Group, School of Biochemistry and Immunology, Trinity College Dublin, Dublin, Ireland; 2 Centre for Microscopy and Analysis, Trinity College Dublin, Dublin, Ireland; 3 Laboratory of Molecular Parasitology, Université Libre de Bruxelles (ULB), Gosselies, Belgium; Technion-Israel Institute of Technology, Israel

## Abstract

Flagellar attachment is a visibly striking morphological feature of African trypanosomes but little is known about the requirements for attachment at a molecular level. This study characterizes a previously undescribed membrane protein, FLA3, which plays an essential role in flagellar attachment in *Trypanosoma brucei*. FLA3 is heavily *N*-glycosylated, locates to the flagellar attachment zone and appears to be a bloodstream stage specific protein. Ablation of the *FLA3* mRNA rapidly led to flagellar detachment and a concomitant failure of cytokinesis in the long slender bloodstream form but had no effect on the procyclic form. Flagellar detachment was obvious shortly after induction of the dsRNA and the newly synthesized flagellum was often completely detached after it emerged from the flagellar pocket. Within 12 h most cells possessed detached flagella alongside the existing attached flagellum. These results suggest that proteins involved in attachment are not shared between the new and old attachment zones. In other respects the detached flagella appear normal, they beat rapidly although directional motion was lost, and they possess an apparently normal axoneme and paraflagellar rod structure. The flagellar attachment zone appeared to be disrupted when FLA3 was depleted. Thus, while flagellar attachment is a constitutive feature of the life cycle of trypanosomes, attachment requires stage specific elements at the protein level.

## Introduction

African trypanosomes are flagellated protozoan parasites that cause sleeping sickness in humans and trypanosomiasis in cattle. The main proliferative forms of the life cycle of these parasites are the long slender form of the mammalian bloodstream and the procyclic form of the midgut of the tsetse fly vector. Trypanosomes possess a single flagellum that has established roles in motility, cell division, organelle positioning and pathogenicity and may also have a sensory role [1]–[11]. The flagellum of trypanosomes contains a classical axoneme with a 9+2 arrangement of microtubules but possesses an additional large, extra-axonemal structure called the paraflagellar rod (PFR) [1]–[4]. Unusually, the flagellum is attached to the cell body of trypanosomes [1], [2], [12]. As it emerges at the posterior end of the cell through an invagination of the plasma membrane called the flagellar pocket, it is bent and attached to the cell body through a specialized domain called the flagellar attachment zone or FAZ. The FAZ is a complex structure, involving cytoskeleton and membrane components of the cell body and flagellum, which runs along the cell surface from the flagellar pocket to the anterior end of the cell body. The distal tip of the flagellum extends beyond this point to give rise to a “free” flagellum. Two prominent features of the FAZ are visible in electron micrographs of the region. First, the FAZ contains an electron dense filament, positioned in a gap in the subpellicular array of microtubules that are located immediately beneath the surface membrane of the cell body. This filament appears as a regularly spaced structure that runs the length of the attachment zone [1], [13]. Filament-like structures also appear to link the PFR to the membrane region closest to the FAZ filament on the flagellum side of the attachment zone. A second feature of the attachment zone is the presence of a special quartet of microtubules located immediately to the left of the FAZ filament when the cell is viewed towards the anterior end. These microtubules originate at the end of the flagellum closest to the basal bodies and run the length of the attachment zone alongside the FAZ filament. This quartet is intimately associated with a sheet of endoplasmic reticulum, which interdigitates between the four microtubules [13].

Flagellar attachment involves novel structures which may represent targets for therapeutic intervention since attachment is essential for growth and motility of the bloodstream form of these parasites. Attachment is of general cell biology interest also as it requires the spatial and temporal co-ordination of structures from the cell body and flagellum across a junction as the growing flagellum is attached to the cell. Although electron microscopy first revealed novel features of the FAZ more than 40 years ago [1], relatively little is known about the proteins and mechanisms involved in flagellar attachment. Several insights have come from the use of monoclonal antibodies that recognize various elements of the FAZ [3], [14]–[18]. One of these monoclonal antibodies (L3B2) binds to the cell body side of the FAZ filament. This monoclonal was subsequently used to screen a cDNA expression library and identified the antigen as a large, repetitive protein containing a 14 residue repeat that was termed FAZ1 [19]. Depletion of FAZ1 in the procyclic form of *T. brucei* resulted in the formation of a compromised FAZ and led to defects in flagellar attachment and cytokinesis [19]. Recently, a protein (CC2D) containing a coiled-coil and CD2 domain was shown to be associated with the FAZ filament, FAZ-associated ER and the basal body in the procyclic form [20]. Depletion of CC2D inhibited the assembly of a new FAZ filament leading to detached flagella and defects in cell growth and morphology. The first membrane protein shown to be involved in flagellar attachment in *T. brucei* was the homologue of a 72-kDa flagellar-adhesion glycoprotein (Gp72) from the related parasite *T. cruzi* [21]. This protein, termed FLA1 for flagellar-adhesion glycoprotein, is expressed in bloodstream and procyclic forms and was found to localize to the flagellum and the flagellar pocket. The location of FLA1, the level of similarity with Gp72 (63%) and the fact that it appeared to be an essential protein in the bloodstream form suggested that FLA1 had a role in flagellar attachment. This role was subsequently confirmed by RNA interference which demonstrated that knockdown of FLA1 caused a defect in flagellar attachment and a failure in cytokinesis [22]. Interestingly, this study also identified a transcript for a very closely related, but as yet uncharacterised, protein (FLA2) that may play a role in flagellar adhesion in bloodstream forms. Finally, a recent flagellar surface proteome study has identified a putative calcium channel (FS179) which localizes to the FAZ and is required for flagellar attachment [23]. To date FLA1 and FS179 are the only membrane proteins that have been located to the FAZ and which have been shown to have a definite role in flagellar attachment. In this study we add another protein, FLA3, to this group by identifying and characterizing a previously unknown membrane protein and demonstrating that it has an essential role in flagellar attachment. The expression and functional requirement for FLA3 appears to be restricted to the bloodstream form which indicates that flagellar attachment requires life cycle stage specific components.

## Results

### Identification of a Gene for Coding for a Protein Present in a Tomato Lectin Binding Fraction Isolated from the Bloodstream form of *T. brucei*

We previously described the screening of a cDNA expression library using polyclonal antibodies raised against a total tomato lectin binding fraction isolated from the slender bloodstream form of *T. brucei* to generate a “mini-library” of cDNAs that potentially code for proteins present in a tomato lectin binding fraction isolated from these cells [24]. Glycoproteins that possess *N*-linked poly-*N*-acetyllactosamine side chains bind to tomato lectin and appear to be associated with the endocytic pathway and flagellar pocket in these cells [25]. Almost half of the cDNAs identified were for hypothetical proteins with no established function. Some of these hypothetical proteins were found in other organisms, while most others (∼ 80%) appeared to be trypanosome specific. One of these trypanosome specific sequences corresponded to a partial cDNA of ∼ 1.4 kb that encoded a putative open reading frame of 344 amino acids (∼1 kb) followed by an apparent 3′ untranslated region of 329 bp (Fig. S1). A subsequent screening of the completed genome of *T. brucei* revealed that this open reading frame corresponded to the *C*-terminal end of a protein encoded by a tandem of two genes with the systematic entry numbers Tb927.5.4570/80. These two genes encode virtually identical proteins (∼ 92% identity) containing 818 residues with a predicted size of ∼ 89 kDa. The sequence variation was restricted to small stretches or individual residues within the *N*-terminal 250 residues of the putative proteins. Analysis of the primary structure predicted the presence of two hydrophobic stretches with the potential to be membrane spanning regions located close to the *N* (residues 22–45) and *C* (residues 751–772) termini of the protein (Fig. S2). The protein was not predicted to possess a cleavable *N*-terminal signal sequence. The putative *C*-terminal membrane spanning region (21 residues) was immediately followed by a stretch of four basic residues which would suggest that the *C*-terminal tail is located on the internal side of the membrane according to the positive inside rule [26]. This analysis was consistent with a topological organization consisting of a large extracellular/luminal region, representing ∼ 90% of the protein, flanked by two transmembrane spans with short regions at the C-terminus (45 residues) and N-terminus (21 residues) located on the internal side of the membrane (Fig. S2). The putative extracellular region was predicted to contain many *N*-glycosylation sites. There was no significant similarity with proteins of known function nor were there significant matches with signatures or motifs in the protein domain databases. A search of the *T. brucei* genome revealed the presence of a related protein (∼ 45% identity) that was encoded by two identical genes with the systematic entry numbers Tb927.8.4050/4100 (Fig. S3). Both genes code for an identical protein of 750 residues with an expected size of 83 kDa. A basic alignment indicated that sequence similarity occurs throughout the proteins but that the protein encoded by Tb927.5.4570/80 contains an insertion of 30 residues (620–650) that is missing in the protein encoded by Tb927.8.4050/100. This latter protein also appears to contain two potential transmembrane spanning regions located close to the beginning (residues 19–43) and end (residues 688–710) of the open reading frame. This arrangement would allow a similar topological organization to that proposed for the related protein encoded by Tb927.5.4570/80. Analysis of the genomes of other kinetoplastids revealed the presence of genes coding for proteins that are clearly related to those encoded by Tb927.5.4570/80 and Tb927.5.4050/100. In the case of other African trypanosomes the level of identity ranged between 40–45% (E value scores >1.0e–175), while in the case of the more distantly related parasites *T. cruzi* and *Leishmania* species the level of identity was lower and ranged between 24–34% (E value scores >1.0e–110).

### Expression Analysis

**Figure 1:**
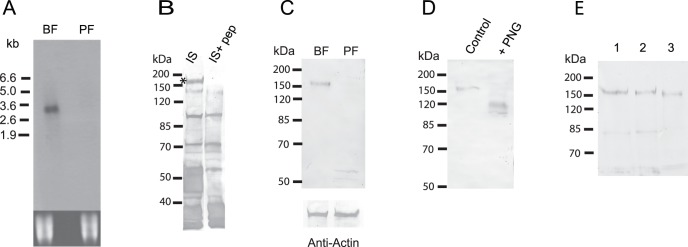
Tb927.5.4570/80 codes for a membrane glycoprotein expressed in the bloodstream form of *T. brucei*. Panel A. Northern blot analysis of 5 µg of total RNA from bloodstream (BF) and procyclic (PF) form of *T. brucei*. The blot was probed with a fragment (∼600 bp) corresponding to the region between nucleotides 1174 to 1792 of Tb927.5.4580 under conditions of high stringency [39]. The lower panel represents the ethidium bromide staining of the RNA as a control for loading. Panel B. Immune serum (IS) raised against a peptide antigen was used to probe blots of total cell lysates of bloodstream forms (5×10^6^ cells per lane). The blots were performed in presence (IS+pep) or absence (IS) of the peptide antigen (5 µg/ml). An asterisk marks a band (∼160 kDa) that was not detected when the peptide antigen was present during the incubation with the immune serum. Proteins migrating between 40–50 kDa were also lost when the peptide antigen was included (see Panel C). Panel C. Affinity purified antibodies were used to probe total lysates (5×10^6^ cells/lane) of bloodstream (BF) and procyclic forms (PF). The lower panel represents the level of actin as a control for loading. Panel D. Resin immobilized affinity purified antibodies were employed to immunoprecipitate the 160 kDa protein from lysates of bloodstream form trypanosomes. The immunoprecipated protein was incubated in the absence (Control) or presence of PNGase F (+PNG) to remove *N*-glycans. Following treatment with PNGase F the samples were subjected to immunoblot analysis using the affinity purified antibodies. Panel E. Trypanosomes were subjected to mechanical disruption by grinding with glass beads. A pellet fraction was isolated from the resultant homogenate by centrifugation and subjected to detergent extraction. The initial homogenate (lane 1), pellet fraction (lane 2) and the detergent soluble fraction isolated from the pellet (lane 3) were subjected to an immunoblot analysis using the affinity purified antibodies. Each lane represents a total of 5×10^6^ cell equivalents.

The relative level of expression of the transcript encoded by Tb927.5.4570/80 was investigated by a Northern blot analysis and revealed significant up-regulation of a transcript (∼3.4 kb) in the bloodstream relative to procyclic form (Fig. 1A). This up-regulation was confirmed by qRT-PCR which gave a relative expression ratio (bloodstream/procyclic form) of 7.7±1.1 and 8.6±0.8 (mean ± SD of three separate determinations) using total RNA and purified mRNA fractions respectively. This level of differential expression was similar to that observed for the bloodstream stage specific glycosylphosphoinositol specific phospholipase C transcript [27], where a relative expression ratio of 10.6±1.9 (mean ± SD of three determinations) was obtained using the same total RNA sample. The same analysis was conducted for the transcript encoded by the related genes Tb927.8.4050/4100 and a relative expression ratio (BF/PF) of 0.21±0.09 (mean ± SD of three determinations) was obtained indicating that this mRNA was five-fold more abundant in the procyclic form. Interestingly, this related transcript was not detected in either form by Northern blotting probably because the blot was probed under conditions of high stringency. Attempts to purify recombinant forms of the protein encoded by Tb927.5.4570/80 to generate specific antibodies were complicated by difficulties in solubilization of the fusion proteins, even those lacking the hydrophobic regions. Therefore, polyclonal antibodies were raised in rabbits against the *C*-terminal 18 amino acids of the putative protein and used to probe total cell lysates. Although significant nonspecific reaction was observed with many proteins using the immune serum, a protein band that migrated with an apparent size ∼ 160 kDa was not detected when the peptide antigen was included with the immune serum during the blotting step (Fig. 1B). Therefore, the immune serum was subject to affinity purification using the peptide antigen immobilized onto an activated resin and the resultant affinity purified antibodies were used in immunoblotting experiments to assess expression at the protein level. The results demonstrated up-regulation of the expression of a protein (∼160 kDa) in the bloodstream relative to procyclic form (Fig. 1C). Two faint bands that migrated just above 50 kDa were also detected in the procyclic but not bloodstream form. These bands probably represent nonspecific cross reaction with unrelated proteins rather than cross reaction with the related protein encoded by Tb927.8.4050/4100 which would be expected to be much larger (∼ 83 kDa). Overall these data were consistent with the results from the transcript analysis and indicated that the protein encoded by the genes Tb927.5.4570/80 undergoes differential expression during the life cycle. Treatment of the immunoprecipitated protein with PNGase F resulted in the disappearance of the 160 kDa band and the appearance of smaller forms of the protein that migrated with apparent sizes between 100–120 kDa (Fig. 1D). This result suggested that the discrepancy between the expected and observed size (89 v 160 kDa) of the protein was due, at least in part, to extensive post-translational modification by *N*-glycosylation. The protein was also predicted to contain membrane spanning regions so detergent fractionation experiments were performed to determine if the protein behaved as a membrane protein. Significantly the protein was recovered almost entirely in the particulate or membrane fraction following mechanical disruption of the cells while subsequent detergent extraction of this fraction resulted in complete solubilization of protein (Fig. 1E). Together these data demonstrated that the gene tandem Tb927.5.4570/80 encoded a previously uncharacterized extensively *N*-glycosylated membrane protein likely to be bloodstream stage specific.

### Localization Studies

**Figure 2:**
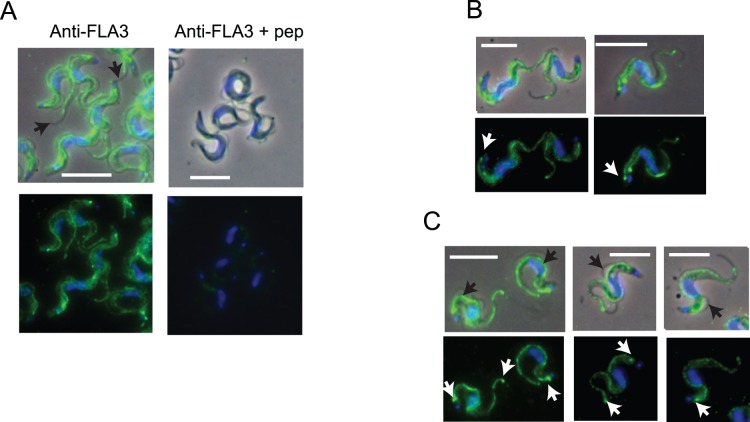
Localization of FLA3 in the bloodstream form of *T. brucei*. Trypanosomes were purified from infected blood, fixed and processed for immunofluorescence using the affinity purified α-FLA3 antibodies. Upper panels present a merge of the fluorescence and phase images and reveal the location of FLA3 (green) and the nucleus/kinetoplast (blue); lower panels present the fluorescence image alone. Bar = 10 µm. Panel A. FLA3 was detected as a punctate pattern of staining along the FAZ. The black arrows in the upper panel (α-FLA3) indicate the absence of FLA3 along the free flagellum. Non specific binding was investigated by including the peptide antigen (10 µg.ml^−1^) (α-FLA3+ pep). Panel B. FLA3 is associated with existing and new (arrows) FAZ. Panel C. FLA3 remains associated with the cell body at regions where the flagellum has become detached (black arrows in the phase/fluorescence merge). The white arrows indicate more pronounced staining of FLA3 frequently observed at the posterior and anterior end of the attachment zone (lower panels).

**Figure 3:**
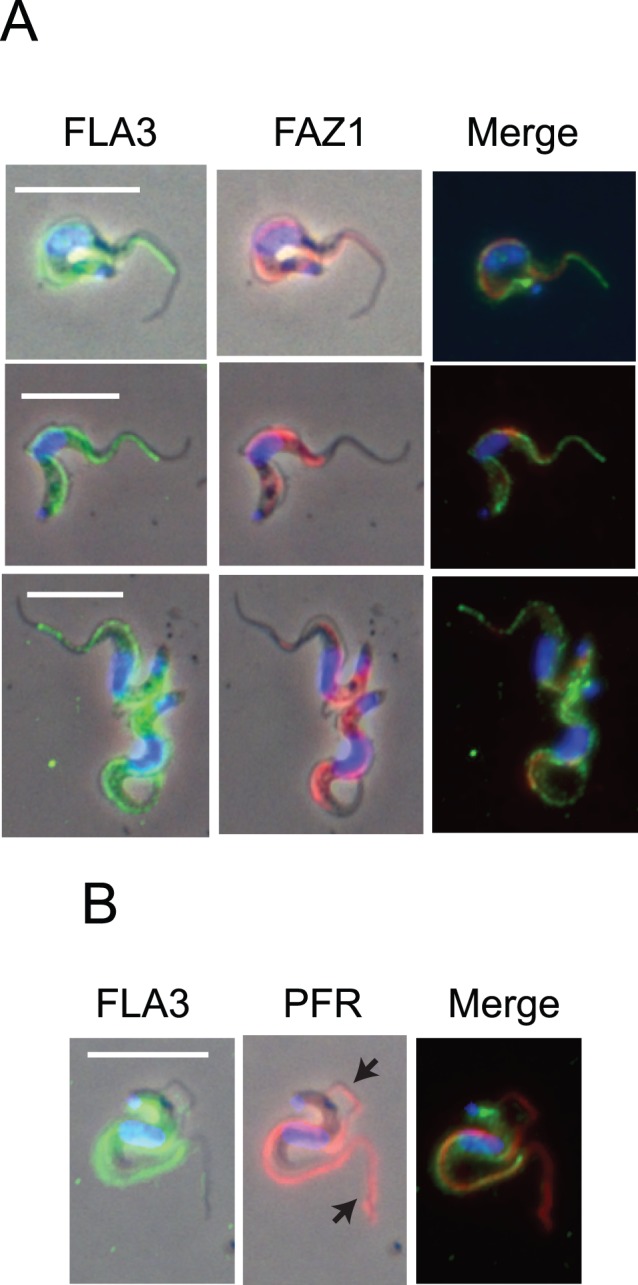
Co-localization of FLA3 with markers for the FAZ and flagellum. Cells were probed using affinity purified α-FLA3 rabbit antibodies and a mouse monoclonal antibody against FAZ1 [19] or PFR2 [18]. The cells were examined using an Olympus Fluoview 1000 confocal microscope. The panels are presented as merges of the phase and the individual immunofluorescence images plus a combined antibody fluorescence merged image. Bar = 10 µm. Panel A. The location of FLA3 (green), FAZ1 (magenta) and the nucleus/kinetoplast stained with DAPI (blue). Panel B. The location of FLA3 (green), PFR2 (magenta) and the nucleus/kinetoplast (blue). PFR2 but not FLA3 is associated with the flagellum and the free flagellum at the anterior end of the cell (black arrow).

The localization of the 160 kDa membrane glycoprotein encoded by Tb927.5.4570/80 was investigated in fixed bloodstream forms by indirect immunofluorescence using affinity purified antibodies. These experiments revealed a strong, punctate fluorescence signal associated with the attachment zone of the flagellum in cells, along with a weaker more diffuse signal that was distributed throughout the cell body (Fig. 2A). The signal associated with the attachment zone appeared to be specific as it was lost when the peptide antigen was included with the primary antibody. The signal associated with the cell body was also lower. Previous studies have demonstrated that FLA1, a membrane glycoprotein required for flagellar attachment, was located in the flagellar pocket and along the attachment zone [21]. Since the 160 kDa protein was also associated with the FAZ it was named FLA3, following the convention adopted for FLA1 & 2 [21], [22], although there was no sequence similarity with either of these proteins. Significantly, FLA3 localized to the attachment zone of the new flagellum as it grew posterior to the existing flagellum but was never found along the free flagellum (Fig. 2B). In many cells FLA3 seemed to be more abundant at the beginning and at the end of the attachment zone (Fig. 2B & C). In cells where the flagellum occasionally became partially detached during the fixation and processing steps FLA3 was always located on the cell body side of the detached region (Fig. 2C). Co-localization studies were performed using monoclonal antibodies against FAZ1, a protein containing a 14 residue repeat associated with the cell body side of the FAZ filament [19] and the paraflagellar rod protein PFR2 [18]. Both FLA3 and FAZ1 exhibited a similar but not identical distribution along the attachment zone. For example, as noted previously FLA3 appears to more abundant at either end of the FAZ and also to extend more toward the anterior end of FAZ than does FAZ1. However, neither protein appeared to be associated with the flagellum, as seen where the flagellum was clearly distinguishable from the cell body, nor was either protein associated with the free flagellum at the anterior tip of the cell (Fig. 3A). The location of both FLA3 and FAZ1 was clearly different to PFR2, which was detected in the flagellum and not the FAZ (Fig. 3B). Taken together, the results from these localization studies were consistent with view that FLA3 was a membrane glycoprotein associated with the cell body side of the flagellar attachment zone.

### FLA3 is Essential in Bloodstream forms of *T. brucei*

**Figure 4:**
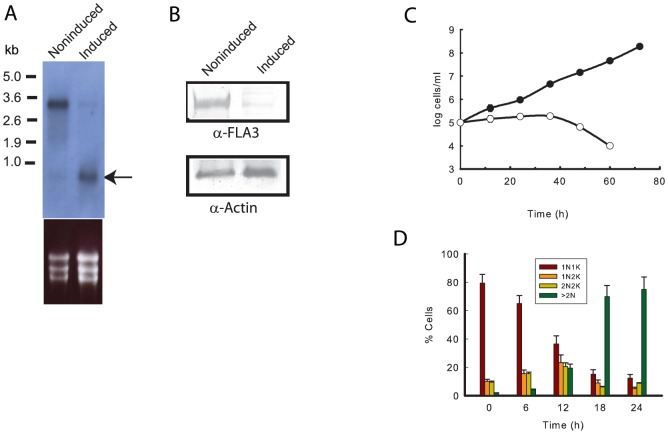
FLA3 is an essential protein in the bloodstream form o*f T. brucei.* Panel A. Northern blot analysis of 15 µg of total RNA from a cloned FLA3 bloodstream RNAi cell line grown under induced or noninduced conditions for 24 h. The probe used for hybridisation was the same fragment employed for RNAi. The lower part of the panel presents the ethidium bromide staining of the RNA as a control for loading. The arrow indicates the presence of the dsRNA (∼ 600 bp) in the induced cells. Panel B. An immunoblot analysis confirmed loss of the FLA3 protein in the induced cells after 30 h induction. Each lane contained 5×10^6^ cells and a loading control was performed with antibodies against trypanosome actin. Panel C. The effect of knock down of FLA3 on the growth of a representative cloned FLA3 RNAi cell line cultured in the presence (○) or absence of tetracycline (•). Panel D. Analysis of the number of nuclei and kinetoplasts per cell in a FLA3 RNAi cell line cultured in the presence of tetracycline. For each time point individual cells were assessed for the presence of kinetoplast and nucleus and scored as 1N1K, 1N2K, 2N2K and cells with clearly more than 2N. The results were expressed as the mean ± SD of four separate surveys of 50 cells.

The functional role of FLA3 was assessed by conditional RNAi using a construct that allowed the tetracycline-inducible expression of a double stranded RNA (dsRNA) common to both genes for FLA3 (nucleotides 1174–1792). Induction of the dsRNA resulted in a decrease of ∼ 80% in the *FLA3* transcript after 18 hours as indicated by the relative expression ratio (induced/noninduced) of 0.19±0.09 determined by qRT-PCR (mean ± SD of three separate determinations). A Northern blot analysis also revealed a significant knockdown of the *FLA3* transcript in the induced cells (Fig. 4A). The same qRT-PCR analysis also confirmed that induction of *FLA3* RNAi had no effect on the levels of the transcript encoded by Tb927.8.4050/4100 (not shown). This result was also consistent with the detection of a single transcript that corresponded to *FLA3* in the bloodstream form and no transcripts in the procyclic form when the region employed for RNAi was used to probe Northern blots (Figs. 1A & 4A). A Western blot analysis confirmed knockdown of FLA3 at the protein level (Fig. 4B). Depletion of FLA3 had a deleterious effect on the growth of bloodstream forms (Fig. 4C). Cell division ceased ∼ 8–12 h after induction of the dsRNA and cell death occurred about 2–3 days later. During this period noninduced cells continued to grow and divide with a doubling time of about 7 hours. The same results were obtained with several independent FLA3 bloodstream RNAi clones. Analysis of the cellular kinetoplast/nucleus ratio indicated that the effect on growth was primarily at the level of cytokinesis, as there was a progressive increase in the number of multi-nucleated cells in the population following induction of the dsRNA (Fig. 4D). A procyclic FLA3 RNAi line was also generated and induction of the FLA3 dsRNA, to knockdown any residual *FLA3* mRNA, had no effect on the growth of these cells (Fig. S4). Taken together, these data were consistent with stage specific expression of FLA3 and demonstrated that FLA3 is an essential protein in the bloodstream form of *T. brucei*.

### FLA3 is Required for Flagellar Attachment

**Figure 5:**
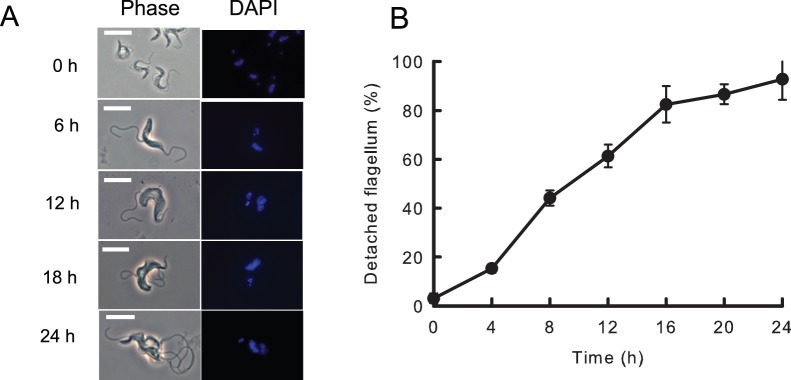
Knockdown of FLA3 leads to flagellar detachment in the bloodstream form of *T. brucei.* Panel A. FLA3 RNAi cells were grown in the presence of tetracycline. Samples were removed, fixed and processed for phase contrast and fluorescence microscopy to assess flagellar attachment (Phase) and DNA content (DAPI) of the cells. Bar = 10 µm. Panel B. At each time point the number of cells with detached flagella was assessed as a % of the total number of cells in the population. The results were expressed as the mean ± SD of four separate surveys of 50 cells.

**Figure 6:**
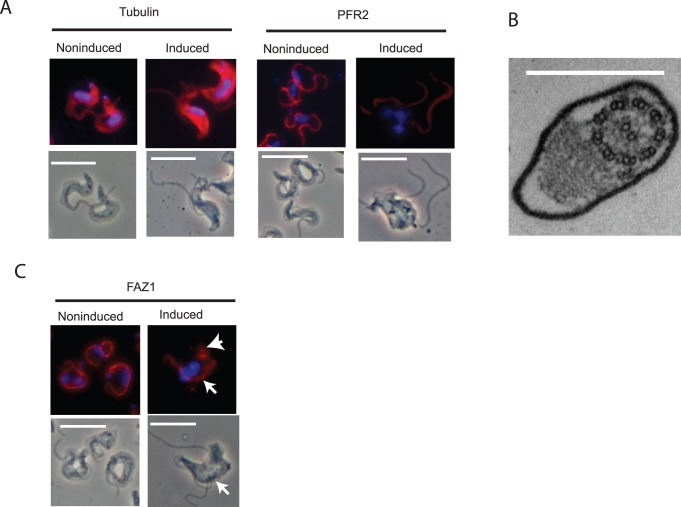
The effect of the loss of FLA3 on the location of proteins associated with the flagellum and flagellar attachment zone. Bloodstream form FLA3 RNAi cells were grown under induced or noninduced conditions for 24 h. Panel A. Immunolocalization of α-tubulin and paraflagellar rod protein 2 (PFR2) in induced and noninduced cells. The upper panels represent the fluorescence image showing location of the protein (red) and nucleus/kinetoplast (blue), while the lower panels represent the corresponding phase image. Bar = 10 µm. Panel B. A sample section through a detached flagellum. Bar = 500 nm Panel C. Localization of FAZ1 in induced and noninduced cells. The upper panels represent the fluorescence image showing location of the protein (red) and nucleus/kinetoplast (blue), while the lower panels represent the corresponding phase image. Bar = 10 µm. The arrowhead indicates apparent aberrant accumulation of FAZ1 at the posterior end of an induced cell which has a single attachment zone containing FAZ1 associated with the attached flagellum (arrow in phase and fluorescence image).

**Figure 7:**
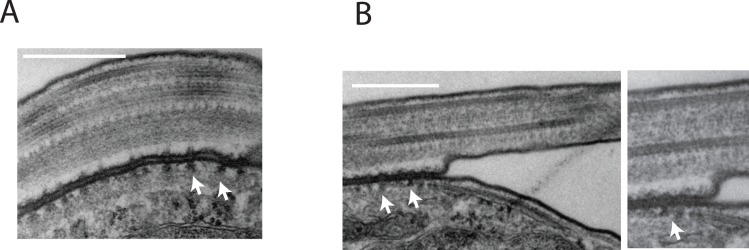
The effect of the loss of FLA3 on the flagellar attachment zone. Panel A. Detail of the flagellar attachment zone in noninduced FLA3 RNAi cells showing the regularly spaced FAZ filament structure (arrow). Panel B. Detail of the flagellar attachment zone in an induced FLA3 RNAi cells at the point where the flagellum became detached reveals the loss of the regularly spaced FAZ filament structure (arrows). The panel on the left presents a close up of the region where detachment occurred. Bar = 500 nm.

Knockdown of FLA3 produced an early and obvious defect in flagellar attachment. Even after an induction of only 6 hours a significant number (∼ 25%) of the cells possessed a detached flagellum (Fig. 5A & B). At this stage the new flagellum, which emerged on to the cell surface posterior to the existing flagellum, was frequently initially attached to the cell body but became detached during subsequent growth of the flagellum (Figs. 5A & S5). Once the flagellum became detached it remained as a detached, free flagellum (Fig. 5A). In many cases the new flagellum never became attached after it emerged from the flagellar pocket posterior to the existing attached flagellum (Fig. S5). The number of cells in the induced population with detached flagella continued to increase so that after 24 hours virtually all cells in the population possessed multiple detached flagella (Fig. 5). The failure of cytokinesis was evident in the increased size, aberrant morphology and multi-nucleate nature of these cells. The detached flagella continued to beat rapidly, although directional motility was lost in these cells (Video S1). Flagellar attachment and cell motility was normal in the noninduced FLA3 RNAi cells (Video S2). During this period the defect in attachment appeared to be restricted to the new flagellum while the existing flagellum remained attached to the cell. In the case of flagella that never became attached, the flagellum still traversed the flagellar pocket and remained tightly associated with the neck region of the flagellar pocket as normal but did not bend and attach to the cell body (Fig. S6). In these cases the flagellar pocket appeared to be of normal size and morphology (Fig. S6B). There was also evidence of normal flagellar pocket function in these cells, such as recruitment of clathrin and formation of coated vesicles. The observation that detached flagella continued to beat and grow suggested that they retained many of the structural features of attached flagella. This view was consistent with the presence of tubulin and PFR2 in all the detached flagella (Fig. 6A). The more intense tubulin staining of the cell body of the induced cells indicated an accumulation of tubulin in cells that had failed to undergo cell division. Frequently, sections occurred through detached flagella in micrographs of knockdown cells and these revealed an apparently normal axonemal and paraflagellar rod structure in the detached flagellum (Fig. 6B). However, FAZ1 appeared to mislocate when FLA3 was depleted as the protein was observed to accumulate at the posterior end of the cells containing multiple detached flagella (Fig. 6C). A single line of FAZ1 still remained associated with the attached flagellum in these cells. These results suggested that FLA3 is essential for the assembly of a new attachment zone and that knockdown of the protein results in mislocalization of key elements of this structure, such as FAZ1. This view was supported by the finding that in cells where the flagellum was initially attached, detachment of the flagellum seems to be accompanied by a loss of the regularly spaced FAZ filament structures (Fig.7). The flagellar membrane along the flagellar side of the FAZ also seemed to be physically closer to the paraflagellar rod from the point at which detachment occurred.

## Discussion

Flagellar attachment is a visibly striking morphological feature of trypanosomes that involves novel structures which are as yet poorly understood at a molecular level. This study describes a previously uncharacterized membrane glycoprotein, FLA3, and demonstrates that it locates to the FAZ and plays an essential role in flagellar attachment. Interestingly, FLA3 appears to be a bloodstream stage specific protein, which distinguishes it from other FAZ proteins, such as FLA1 and FAZ1 that appear to be involved in attachment throughout the life cycle. Thus, while flagellar attachment is a constitutive feature of trypanosomes, and structures such as the FAZ filament and FAZ associated quartet of microtubules are always present, it seems that attachment requires stage specific elements at the protein level. There are probably other stage specific proteins involved in flagellar attachment. For example, the transcript for the FLA3 related protein identified in this study (Tb927.5.4050/100) is more abundant in the procyclic form. Given the level of similarity (45%) and the structural features it shares with FLA3 it seems reasonable that this FLA3-related protein may have a role in flagellar attachment during the procyclic stage of the life cycle. Furthermore, knockdown studies on FLA1 in bloodstream forms [22] were complicated by the presence of a bloodstream stage specific transcript that encoded a closely related protein called FLA2 (61% identical to FLA1). This *FLA2* mRNA was simultaneously ablated in the FLA1 RNAi experiments so it was not possible to conclude unequivocally whether FLA1 and/or FLA2 were required for attachment. Consequently, FLA2 may represent another bloodstream stage specific protein involved in flagellar attachment. Two other interesting features of FLA3 appear to be its relatively higher abundance at the beginning and end of the FAZ as well its punctate distribution along the attachment zone. The latter feature has been observed for other FAZ associated proteins such as CD10 and DOT1 [15] and proteins identified in the flagellar surface proteome [23]. While both FLA3 and FAZ1 locate to the cell body side of the FAZ they probably have different roles in attachment since FLA3 is a membrane protein, while FAZ1 is a detergent insoluble cytoskeletal component of the FAZ filament, a structure which extends into the cytoplasm. Attempts to obtain higher resolution localizations of FLA3 using immunogold techniques have not been successful so far possibly because fixation and resin embedding affects antibody recognition of the antigen target.

Conditional knockdown of FLA3 revealed a number of interesting features of flagellar attachment in the bloodstream form. First, attachment is not required for flagellar growth, a feature that was also observed in FAZ1, CC2D, FLA1 and FS179 knockdown experiments [19], [20], [22], [23]. The detached flagella in FLA3 depleted cells continue to beat which indicates that most flagellar components are probably present. Indeed, the use of RNAi to detach flagella has been exploited to generate a flagellum surface and matrix proteomes [23]. Second, depletion of FLA3 affected the attachment of the new but not the existing flagellum, at least initially. A similar selective effect was observed when other FAZ proteins were depleted [19], [20], [22], [23]. This selectivity suggested that constituents of the FAZ cannot be exchanged between the existing and new attachment zone, which probably explains why the attachment defect is visible so soon after induction of the FLA3 knockdown. Finally, detachment of the flagellum appeared to be accompanied by a mislocalization of FAZ1 with an apparent accumulation of the protein at the posterior end of the cell. In addition, there appears to be a loss of the regularly spaced FAZ filament structures from the point where the flagellum became detached. These findings suggested that FLA3 depleted cells cannot assemble a new FAZ filament structure. At present it is unclear how FLA3 might affect the formation of the FAZ filament but it may involve interactions with the short regions of the protein that are likely to be located on the cytoplasmic side of the surface membrane.

In the case of FAZ1 procyclic RNAi cells the defect in attachment appears less severe than that observed for FLA3 bloodstream RNAi cells. The most common defect observed in the FAZ1 knockdown cells was partial detachment and the appearance of a loop of detached flagellum, usually in the central portion of the cell, which was flanked by attached regions [19]. It was proposed that loss of FAZ1 leads to weak and faltering assembly of the FAZ behind the growing flagellum, which in turn gives rise to areas of flagellar detachment. However, in procyclic forms the tip of the growing new flagellum is physically attached to the existing flagellum by a structure called the flagellar connector, which is thought to guide and position the new flagellum along the cell surface [28], [29]. Indeed, the detached flagellum in FLA1 depleted procyclic cells has been observed to form a loop as it remains attached to existing flagellum via the flagellar connector [28]. However, the flagellar connector appears to be absent in bloodstream forms, which may explain why the flagellum becomes completely detached in the FLA3 depleted cells. The effect of the loss of FAZ1 in bloodstream forms was not investigated but it would be interesting to see if the defect in attachment is more severe in these cells. The level of knockdown may also have affected the severity of the defect in FAZ1 RNAi cells, as completely detached flagella were observed when CC2D was depleted by 90% in procyclic forms [20]. In general bloodstream form parasites seem more sensitive to flagellar defects than the procyclic form [8]. Treatment with non-ionic detergents has been reported to cause flagellar detachment in bloodstream but not procyclic forms [30]. In fact, while flagellar attachment is clearly essential for cell division in the bloodstream form, this may not to be the case for procyclic cells. For example, expression of the *T. cruzi* protein Gp72, which is related to FLA1, appears to lead to a defect in flagellar attachment but the cells continue to grow albeit more slowly [22]. It was proposed that GP72 might interfere with the attachment function of FLA1 without affecting its function in cytokinesis. However, cell division and polarity always seem to depend on the assembly of a FAZ and a FAZ filament so it would be interesting to know if cells expressing Gp72 can still assemble these structures.

Modification by *N*-glycosylation is a feature of two membrane proteins known to be required for flagellar attachment. In FLA3 *N*-glycosylation is extensive and may represent 50% of the mature protein (∼ 160 kDa), while in FLA1 the modification is less extensive with ∼ 20 kDa of the mature protein (∼ 100 kDa) due to *N*-glycans. Is it possible that these *N*-glycans might have a role in flagellar attachment? This proposal has been advanced by Turnock *et al.* [31] who found that knockdown of GDP-fucose biosynthesis in *T. brucei* caused flagellar detachment. It was suggested that glycans associated with proteins involved in flagellar attachment might contribute to the process through carbohydrate/carbohydrate interactions, for example as observed for the fucose containing Lewis X glycans involved in integrin activity in stem cells [32], or via interaction with a putative lectin. Glycans containing poly-*N*-acetyllactosamine feature in some of these proposed interactions [33], [34] and proteins that bind to tomato lectin contain such glycans [35]. However, tomato lectin labels the flagellar pocket and endocytic/lysosomal compartments but not the FAZ in fixed cells [25]. Consequently, if poly-*N*-acetyllactosamine containing glycans are associated with FAZ proteins, possibly FLA3, they may not be free to bind tomato lectin perhaps because they are already associated with carbohydrates and/or lectins. All the putative *N*-glycosylation sites in FLA3 are located between the two potential membrane spanning regions so the presence of *N*-glycans requires that this region be on the external side of the surface membrane and the *C*-terminal tail must be cytoplasmic. As noted previously, FLA3 may be arranged with both membrane spanning regions located in the same membrane thus allowing the large glycan-containing region face the flagellar membrane (Fig. S1). Alternatively, the *N*-terminal membrane spanning region may insert into the flagellar membrane so that FLA3 spans the junction of the surface and flagellar membranes. Interestingly FLA3 has been detected as a minor component in a flagellar surface proteome [22] but on the other hand it was always found on the cell body side of those regions where the flagellum had become detached during fixation and processing. Clearly, further studies will be required to determine the surface organization of FLA3. Recently, a possible flagellar membrane protein required for attachment has been identified [22]. This protein (FS179) appears to be a calcium channel which fits with the proposal, originally advanced in the first detailed description of the FAZ, that Ca^2+^ might be involved in attachment [1].

## Methods

### Ethics Statement

The worked described in this study involved the use of laboratory rats obtained from the BioResources unit, which is the licensed facility at Trinity College Dublin. The work was approved by the Bio Resources Ethics Review committee at Trinity College Dublin and was performed under a certificate A licence issued by the Minister for Health and Children under the Cruelty to Animals Act as amended by European Community Directive 86/609/EC. The approved protocol was as follows: the animals were inoculated with a low dose of the protozoan parasite *Trypanosoma brucei*. These parasites grow in the bloodstream of the animal. After three days the animal was given a general anaesthesia and the entire blood volume was withdrawn by aortic puncture resulting in death. The inoculation step is simple and was performed by trained personnel and caused no obvious distress or suffering. During the infection period rats exhibit no signs of distress or changes in feeding or behaviour. The procedure is standard in the field of parasitology. The blood is withdrawn only under conditions of general anaesthesia and the volume is such that the animal does not recover. The animals were maintained and all procedures were conducted in the BioResources Unit.

### Bioinformatics

Protein and nucleotide sequences were identified BLAST searches of the GeneDB database (http://www.genedb.org). Analysis of open reading frames was performed using the series of sequence analysis programmes available on the ExPASy Bioinformatics Resource Portal (http://expasy.org/).

### Antibodies

A specific peptide (RVSNIGVPLT DGKGTTAP) from FLA3 was coupled via an added *N*-terminal cysteine residue to a carrier protein (KLH) and used to immunize rabbits. At various intervals the immune response was determined by an ELISA using the peptide antigen. A peptide affinity resin was generated by attaching the peptide to Sulpho-Link resin (Pierce) via the N-terminal cysteine after treatment of the peptide with immobilized TCEP disulfide reducing gel (Pierce). The resin (approximately 1 ml) was placed into a column and washed with 10 volumes of column buffer (Tris-Cl 20 mM, NaCl 100 mM, pH 7.5). Rabbit immune serum was dialysed at 4°C overnight against column buffer and then cycled over the affinity resin in a closed loop at a flow rate of 0.2 ml overnight. The following day the column was washed with coupling buffer to remove non specific antibodies. Specifically bound antibodies were eluted by washing the resin with 10 column volumes of glycine (100 mM, pH 2.8) followed by 10 volumes of coupling buffer. Fractions (1.8 ml) were collected into tubes containing 0.2 ml of Tris-Cl (1 M, pH 7.5). Those fractions containing antibodies eluted from the resin at the low pH step were dialysed against Hepes buffer (10 mM, NaCl 150 mM, pH 7.5). The antibodies were stored at −80°C. Mouse monoclonal antibodies against FAZ1 (L3B2) and the paraflagellar rod protein 2 (L8C4) were gifts from Prof. K Gull (Dunn School of Pathology, University of Oxford, UK). Monoclonal anti-α-tubulin antibodies (DM1A) were obtained from Sigma.

### Cell Culture

Parental MITat 1.1 bloodstream cells were isolated from infected rats as described elsewhere [36]. Bloodstream cells (strain 328.114) were grown in HMI-9 media containing 10% fetal calf serum and G418 (2.5 µg.ml^−1^) [37]. Procyclic cells (strain 29.13) were grown in SDM-79 containing 10% fetal calf serum and G418 (25 µg.ml^−1^) and hygromycin (25 µg.ml^−1^) as described previously [38].

### RNAi Constructs

A fragment (∼ 600 bp) from the predicted open reading frame of FLA3 was amplified using the following primers GTGAGGGTGGATCCCGATACATGGG and CCTTACCTCGAGCAGTCGCAGCAG and cloned into the p2T7-177 RNAi vector using the restriction sites Xho I and BamH I (sites underlined). The region selected for FLA3 RNAi was chosen using a combination of BLASTn analysis and the Trypanofan RNAit site to select a region unlikely to affect the transcript for the FLA3 related protein (Tb927.5.4050/100). The construct was linearized with Not I and used to transform the RNAi parental cell lines using the Amaxa parasite nucleofection kit (Lonza) [39]. After phleomycin selection (2.5 µg.ml^−1^) expression of the dsRNA was induced by addition of tetracycline (1 µg.ml^−1^).

### Relative Quantification of the *FLA3* Transcript

The relative level of expression of the *FLA3* mRNA was estimated by quantitative reverse transcriptase PCR (qRT-PCR) using the Brilliant® SYBR Green qRT-PCR Master Mix Kit (1-step) from Stratagene. The analysis was conducted using total RNA isolated using the Stratagene Absolutely RNA purification kit. Typically the reaction (25 µl) contained total RNA (50 ng), the appropriate concentration of SYBR Green Master Mix (Stratagene) and forward and reverse primers. The primers were designed using Beacon Designer (Premier Biosoft) and for FLA3 were TACTATGCGTGCCTTAACTC (forward primer) and CATCCTCCGTAAGACTTGC (reverse primer), for the FLA3 related gene (Tb927.5.4050/100) were GACCACCTGAACAACGATACC (forward primer) and ACGATGAGGACGGCTACG (reverse primer) and for actin were ATGAGCAAGCGATGATGG (forward primer) and CAACTCGTTATAGAAGGTATGG (reverse primer). The assays were optimised for primer concentration, PCR reaction efficiency, precision, sensitivity and production of a single amplicon. The levels of TbMyo1 RNA were normalized against actin mRNA and the relative quantification was calculated by the ΔΔCt method and expressed as a ratio of induced to noninduced or of bloodstream to procyclic form as described previously [40].

### Immunoblotting

Membranes were blocked in Tris buffer saline (Tris-Cl 25 mM, NaCl 150 mM, pH 7.5) containing 5% milk powder, 2.5% fetal calf serum and 0.5% Triton X-100. Affinity purified FLA3 antibodies and trypanosome anti-actin antibodies were used at dilutions of 1/100 and 1/1000 respectively in the blocking reagent. For fractionation experiments the cells washed with a Tes-sucrose buffer composed of sucrose (0.3 M), KCl (5 mM), EDTA (1 mM), phenylmethylsulfonyl fluoride (0.2 mM), leupeptin (40 µg/ml), PMSF (0.2 mM), E-64 (10 µM), TLCK (50 µM), TPCK (50 µM), dithiothreitol (2.3 mM), and Tes (25 mM, pH 7.5). The packed pellet of cells was transferred to a chilled mortar. An equivalent amount of cooled glass ballotini beads (Sigma type III, 150–200 microns) was added to the cells followed by grinding with a cold pestle until examination using a phase-contrast microscope showed that up to 80% of the cells had been disrupted. After a 3–6 fold dilution of the resulting paste with Tes-sucrose buffer the beads were removed from the homogenate by centrifugation at 100 ***g*** for 3 min and a particulate fraction was collection by centrifugation at 21,000×g for 30 min at 4°C.

### Immunofluorescence

Cells were purified from infected rat blood using DEAE chromatography, washed and resuspended (10^7^ cells.ml^−1^) in iso-osmotic PSG (pH 8.0) containing NaCl (44 mM), KCl (5 mM), Na_2_HPO_4_ (57 mM), NaH_2_PO_4_ (3 mM), glucose (10 mM), sucrose (70 mM). The cell suspension was mixed gently by inversion several times with an equal volume of freshly prepared paraformaldehyde (6%, w/v) in phosphate buffered saline (PBS) adjusted to pH 7.5. The suspension was incubated on ice for 10 min. After washing (600×g for 8 min at 4°C) in iso-osmotic PSG buffer the fixed cells were suspended at 2×10^7^ cells.ml^−1^ and applied to poly-lysine coated slides. Culture cells were harvested by centrifugation (1500×g for 5 min at 4°C) and resuspended in iso-osmotic PSG and fixed and processed as described above. After attachment of the cells the slides were washed with PBS containing glycine (10 mM) followed by another wash with PBS. The fixed cells were blocked by incubation in PBS containing BSA (1%), FCS (5%) and (0.25% Triton X-100) for 3 hours or overnight at 4°C. Primary antibodies were prepared in the blocking buffer. For co-localization studies mouse monoclonal antibodies [18], [19] against FAZ1 (L3B2) and PFR2 (L8C4) were diluted 1/4 and 1/40 respectively. Incubation times were 1 hr at room temperature or overnight at 4°C. The slides were then washed with PBS and then incubated with Alexa-labelled secondary antibodies (Molecular Probes) typically diluted 1/1000 in the blocking buffer. The cells were incubated with the secondary antibodies for 1 h at 4°C, and then washed three times with PBS. The cells were mounted in Pro-Long Gold anti-fade reagent containing 4′,6-diamidino-2-phenylindole (DAPI) from Molecular Probes and examined using a Zeiss Axiovert 100 fluorescence microscope equipped with an AxioCam HRc camera or an Olympus Fluoview 1000 confocal microscope equipped with the supplied three standard confocal photomultiplier detectors. The images were captured and processed with the FV1000 image software or Zeiss AxioVision V4.8 software.

### Electron Microscopy

Bloodstream forms were fixed in culture media using electron microscope grade glutaraldehyde in 0.1M cacodylate buffer (pH 7.2). The final concentration of glutaraldehyde was 2.5% (v/v) and the cells were fixed for 1 h at room temperature with frequent inversions. The cells were processed from transmission electron microscopy as described previously [39]. The sections were viewed using a Tecnai 10 electron microscope and the images were captured with a MegaView II camera and processed with AnalySIS (Gmbh) and Adobe Photoshop software. For scanning electron microscopy the cells were fixed using Hepes buffered glutaraldehyde (final 2.5%), attached to glass slides and then dehydrated using a series of alcohol solutions increasing in concentration from 10% to 100%. They were then subjected to critical point drying (CPD). The dry samples were then mounted on carbon tabs and gold sputter coated. A Tescan Mira XMU SEM with a secondary electron detector was used to image the sample. Images were taken at 5 KV and at various magnifications.

## Supporting Information

Figure S1 **Nucleotide sequence of a partial cDNA identified by screening expression libraries with antibodies against a total tomato lectin binding fraction isolated from bloodstream forms of** ***T. brucei.*** Panel A. The partial cDNA (1,364 bp) identified in a mini-library of cDNAs isolated by screening expression libraries with antibodies generated against a tomato lectin binding fraction of proteins isolated from bloodstream forms of *T. brucei*. The sequence identified lacked a 5′minexon (common to all mature cDNAs from *T. brucei*) or poly A tail and was likely to represent a partial cDNA. A putative stop codon in fame with a large open reading frame is underlined. Panel B. Analysis of the sequence in panel predicted the presence a large open reading frame (5′ to 3′, frame 1) of 334 residues (∼36 kDa) in frame with the stop codon identified in panel A. Stop codons are indicated and methionine residues are in bold.
(PDF)

Figure S2 **Domain structure of Tb927.5.4570/4580.** Panel A. The full open reading frame (Tb927.5.4580) was identified after interrogation of the geneDB database using the partial cDNA described in Fig.S1. The hydrophobic spans are underlined and the putative N-glycosylation sites shown in red. A basic region located immediately C-terminal to the second putative membrane spanning region is boxed. The potential N-glycosylation sites are all located between the putative membrane spanning regions. The hydrophobic spanning regions and potential N-glycosylation sites were identified using the series of sequence analysis programmes available on the ExPASy Bioinformatics Resource Portal (http://expasy.org/). Panel B. The open reading frame in panel A can be organized as a surface membrane protein with two membrane spanning regions. The model shows short N-and C-terminal regions located on the internal or cytoplasmic face with a large extracellular domain, containing the glycosylation sites, that is flanked by the membrane spanning regions (see Discussion).
(PDF)

Figure S3 **Sequence of Tb927.8.4050 and alignment with Tb927.5.4580.** Panel A. Sequence of Tb927.8.4050 a protein identified by BLAST analysis to be related to Tb927.5.4580/70. two potential membrane spanning regions are underlined and a basic region located immediately C-terminal to the second putative membrane spanning region is boxed. Panel B. A CLUSTAL alignment of the proteins predicted to be encoded by genes Tb927.5.4580 and Tb927.8.4050 showing identical and conserved residues (*, :). The alignment reveals an inserted in Tb927.5.4580 that is missing in Tb927.8.4050.
(PDF)

Figure S4 **Knockdown of FLA3 does not affect the growth of procyclic forms of T. brucei.** Knock down of residual *FLA3* mRNA had no effect on the growth of a cloned FLA3 RNAi procyclic cell line cultured in the presence (○) or absence of tetracycline (•). A decrease in the residual FLA3 transcript level was confirmed by qRT-PCR which gave a relative ratio (induced/noninduced) of 0.53±0.23 (mean ± SD of four determinations).
(PDF)

Figure S5 **Analysis of flagellar detachment in FLA3 bloodstream RNAi cells using scanning electron microscopy.** The cells were fixed at various times after the induction of the FLA3 dsRNA and processed for analysis by scanning electron microscopy. At the zero time the cells had the typical long slender morphology with the flagellum (black arrow) emerging from the flagellar pocket at the posterior end of the cell and remained attached along the entire cell body until it extended as a free flagellum at the anterior end of the cell. After 12 hours most of the cells had a detached flagellum (white arrow) but also retained an attached flagellum (black arrow). In many cases the new flagellum was fully detached and emerged from the cell posterior to the existing flagellum which remained attached to the cell body. At 18 hours post induction many of the cells possessed multiple detached flagella (white arrow) but still retained an attached flagellum (black arrow). The failure of cytokinesis was obvious at this stage by the abnormal morphology and large size of these cells. Bar = 10 µm.
(PDF)

Figure S6 **Flagellar pocket region of a detached flagellum.** The cells were cultured for 24 h in the presence of tetracycline, fixed and processed for transmission electron microscopy. Panel A. Section through the flagellar pocket of a non induced FLA3 RNAi cell. The flagellum is tightly associated with the neck of the flagellar pocket (FP). After emerging from the pocket the flagellum is bend and attached to the cell surface. The regularly spaced structure of the FAZ filament is clearly visible along the attachment zone (arrows). Panel B. Section through the flagellar pocket of a completely detached flagellum. The flagellum remains tightly associated with the neck of the pocket (boxed region) but fails to attach to the surface and extents as a completely free flagellum. The flagellar pocket possesses has a normal morphology. The arrow indicates apparent formation of coated vesicles which is indicative of endocytic activity. The boxed region is shown on the left. Bar = 1µm.
(PDF)

Video S1 **Detached flagella continued to beat but directional motility was lost.** Bloodstream form FLA3 RNAi cells were grown in the presence of tetracycline for 18 h. The images were captured using phase contrast microscopy.
(AVI)

Video S2 **Flagellar attachment and motility in non induced FLA3 RNAi cells.** Bloodstream form FLA3 RNAi cells were grown in the absence of tetracycline. The images were captured using phase contrast microscopy.
(AVI)
