# Supplementary material for: Identification and Characterization of a Stage Specific Membrane Protein Involved in Flagellar Attachment in Trypanosoma brucei
Source: PLoS One. 2013 Jan 15;8(1):e52846. doi: 10.1371/journal.pone.0052846 (PMC3546053; doi:10.1371/journal.pone.0052846)
Supplement: Figure S1 — Nucleotide sequence of a partial cDNA identified by screening expression libraries with antibodies against a total tomato lectin binding fraction isolated from bloodstream forms of T. brucei. Panel A. The partial cDNA (1,364 bp) identified in a mini-library of cDNAs isolated by screening expression libraries with antibodies generated against a tomato lectin binding fraction of proteins isolated from bloodstream forms of T. brucei. The sequence identified lacked a 5′minexon (common to all mature cDNAs from T. brucei) or poly A tail and was likely to represent a partial cDNA. A putative stop codon in fame with a large open reading frame is underlined. Panel B. Analysis of the sequence in panel predicted the presence a large open reading frame (5′ to 3′, frame 1) of 334 residues (∼36 kDa) in frame with the stop codon identified in panel A. Stop codons are indicated and methionine residues are in bold. (PDF) [file pone.0052846.s001.pdf]

A

TTCTCTTTGATATACGCTGACAAGCCCATAACCTTTGGTAGTGACGTTGCGGAGAATGTCACTGCAGTA
AAGCTTTTGATGCCTCATTCATTCAAGAACGCAACGACACCTAAGCAGCTATCGGCTGCGAACCTCACT
GACTTTGCGCACAACTTGGTCAAGGACTTGCGTGCTTCAGACACTCGTGTCGATATCACATTCCCCGAT
CCCCCGTTTAATTTTTCAGCTGTTGTCCCTGAACGTGAGCAGGAAGTACGTTGGTTTGTCCATGGGAAA
GTAATGAAGCAGCTTGAGATTTGTGAGAGACTTGGTAGCCAGGGCGATGCGGCCGTTATTGCAGCTGCA
GCTGCTGCGACTGCTCGAGGTAAGGCCAATGTCACATTGAATACCAGCGGAGTCAAGGCAAATGATACA
GGGGTTGGTCCCAACACCACAAATACCGCTGGTGGGGCCAATACTACTGCTAATGTTGTCGCTAATGGC
ACTGCCAATGTTATTGTGAATCCCTCCACCAATGCTACTCCAACTGGAACCACCAATGCTAGTGTCACT
AATACTACCGAGAGAGCTGTTCCTGTTGTTGCCCCTACTCAGCCTTCTAATGGATATGCGGAGTGTCGA
TCCGCTATAACCAACCGCACGGAAACACAGAACATGGAACCACCTTACGACAGGAAACACCGATATGAG
GTATTCCTACCTAAAAAGTATGACTTCAATGTGTCATGGTGCGTGGACATCATTGACTGGCGTGACTTG
GACGAGATGTTGAATAACCGCACAGATGAGGTGGTGGAAAAGTCCCTTTCATGGTGCGGCCACGGTTGC
ATTATTGCGTTTGCGGTGGTAGGTTCCCTGATTGCTGCGTGTCTAGTGGTGCTGGCTGTGGTGTTGACA
TCGAAGAGGAGGCGTCTTGCTGCTGTGGTTGCTCCACCGCGCCCAAAGTTTGTATCAACAGTGGAAGAT
GATGAAGAAGATCGTGTATCAAACATCGGTGTGCCACTGACCGATGGGAAGGGAACCACCGCA**CCGTAA**
GTCACGTGCGTTGTAGCTGTATGTGTAACATCACATTCCTTGTGTTTGTGTGGTGTGTTTGCATGCATA
GAATATAATCGGACTAGAAAACACTTTATTTGAGCGTACCCCTGTCCTTGAGTGACACTCTCAGGGGAT
GACGCTGCTTAACATTGTGGAGAGGAAGAGACACAACAGAGAGTAGAGTAATATGTTTGTGTGCAAGAG
ATAATAGAAAGGAGGGGGGGGGGTGGTGTGACTCTGTGTCTTGCTTATGTTATTACCTTCCCTCACTGT
TTCCCCCCCCCCCACATACACACGTACCGGAATCCGTAATATACACGGTATTT

B

F S L I Y A D K P I T F G S D V A E N V T A V K L L **M** P H S F K N A T
T P K Q L S A A N L T D F A H N L V K D L R A S D T R V D I T F P D P
P F N F S A V V P E R E Q E V R W F V H G K V **M** K Q L E I C E R L G S
Q G D A A V I A A A A A A T A R G K A N V T L N T S G V K A N D T G V
G P N T T N T A G G A N T T A N V V A N G T A N V I V N P S T N A T P
T G T T N A S V T N T T E R A V P V V A P T Q P S N G Y A E C R S A I
T N R T E T Q N **M** E P P Y D R K H R Y E V F L P K K Y D F N V S W C V
D I I D W R D L D E **M** L N N R T D E V V E K S L S W C G H G C I I A F
A V V G S L I A A C L V V L A V V L T S K R R R L A A V V A P P R P K
F V S T V E D D E E D R V S N I G V P L T D G K G T T A P **Stop** V T C
V V A V C V T S H S L C L C G V F A C I E Y N R T R K H F I **Stop** A Y
P C P **Stop** V T L S G D D A A **Stop** H C G E E E T Q Q R V E **Stop** Y V C
V Q E I I E R R G G G G V T L C L A Y V I T F P H C F P P P H I H T Y
R N P **Stop** Y T R Y
