# Supplementary material for: Identification and Characterization of a Stage Specific Membrane Protein Involved in Flagellar Attachment in Trypanosoma brucei
Source: PLoS One. 2013 Jan 15;8(1):e52846. doi: 10.1371/journal.pone.0052846 (PMC3546053; doi:10.1371/journal.pone.0052846)

A

```
MCFIFGVEMS NLAKRPMSLR KLPQLLLLIM IGIAFVAVEC IGAPVKLPRR VDTVAGQFGF
DGTTDGSSNV SMLSSPYALC RGRTNDEILV GSSNSFRNYS RKTKETGTFL RGGPTGGLVS
ADAKISKPRS CVRRGSGNHT IIYFVDDQNG LKYINDNEIQ HVTVGNGLSL TSVAIYEKDL
YVTDQNNKSV WRCNVGGAGK PQNCEEKKFT GLTFTAKPEG IAVTSKGIFV AARDSSNKGA
LLWLDMNGGG SKGNVSGGFV DVFSTESGML YAATEKELYT VTATGSAFSV TSFAGKNTSS
CYSHANGEDI VLCDNSRLLV IEEYEMYVTS KEKHTMRALT LPPVNLTAIF RGRPAPVGYP
NTTIMEQFVA SLTEDVNKAL GTNDSYVDPD SVRVDPDTWE TNFTVFVQQT RFDNTTEEKL
RSLTYTQTDK TVDEYYGLTD EYVYIDTVLV PFCDDASLVT IQRALAREAG RALNFSLIYA
DKPITFGSDV AENVTAVKLL MPHSFKNATT PKQLSAANLT DFAHNLVKDL RASDTRVDIT
FPDPPFNFSA VVPEREQEVR WFVHGKVMKQ LEICERLGSQ GDAAVIAAAA AATARGKANV
TLNTSGVKAN DTGVGPNTTN TAGGANTTAN VVANGTANVI VNPSTNATPT GTTNASVTNT
TERAVPVVAP TQPSNGYAEC RSAITNRTET QNMEPPYDRK HRYEVFLPKK YDFNVSWCVD
IIDWRDLDEM LNNRTDEVVE KSLSWCGHGC IIAFAVVGSL IAACLVVLAV VLTSKRRRLA
AVVAPPRPKF VSTVEDDEED RVSNIGVPLT DGKGTTAP
```

B

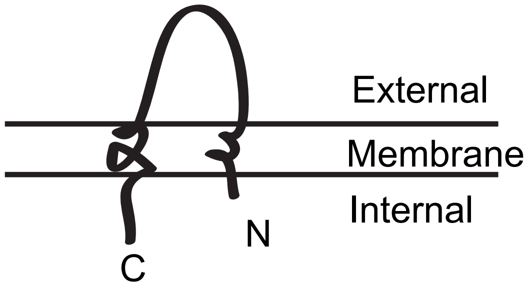

Supplement: Figure S2 — Domain structure of Tb927.5.4570/4580. Panel A. The full open reading frame (Tb927.5.4580) was identified after interrogation of the geneDB database using the partial cDNA described in Fig.S1. The hydrophobic spans are underlined and the putative N-glycosylation sites shown in red. A basic region located immediately C-terminal to the second putative membrane spanning region is boxed. The potential N-glycosylation sites are all located between the putative membrane spanning regions. The hydrophobic spanning regions and potential N-glycosylation sites were identified using the series of sequence analysis programmes available on the ExPASy Bioinformatics Resource Portal (http://expasy.org/). Panel B. The open reading frame in panel A can be organized as a surface membrane protein with two membrane spanning regions. The model shows short N-and C-terminal regions located on the internal or cytoplasmic face with a large extracellular domain, containing the glycosylation sites, that is flanked by the membrane spanning regions (see Discussion). (PDF) [file pone.0052846.s002.pdf]
