# Supplementary material for: Identification and Characterization of a Stage Specific Membrane Protein Involved in Flagellar Attachment in Trypanosoma brucei
Source: PLoS One. 2013 Jan 15;8(1):e52846. doi: 10.1371/journal.pone.0052846 (PMC3546053; doi:10.1371/journal.pone.0052846)
Supplement: Figure S3 — Sequence of Tb927.8.4050 and alignment with Tb927.5.4580. Panel A. Sequence of Tb927.8.4050 a protein identified by BLAST analysis to be related to Tb927.5.4580/70. two potential membrane spanning regions are underlined and a basic region located immediately C-terminal to the second putative membrane spanning region is boxed. Panel B. A CLUSTAL alignment of the proteins predicted to be encoded by genes Tb927.5.4580 and Tb927.8.4050 showing identical and conserved residues (*, :). The alignment reveals an inserted in Tb927.5.4580 that is missing in Tb927.8.4050. (PDF) [file pone.0052846.s003.pdf]

A

```
MPLWKQTNCE VETMNVREVV GTVHLGYVSQ MLLLVATVAT IVVRSGAAPI ELKRHVTTVA
GKYGHIGDKD GFPGMSELSS PHAMCRGRNS DEILLGTVDR FRAFSRKNRE TTTITAWETD
EDQKSGSRSV KVDKPRACVQ WTVGGSTFVY FVESMGEVKY FKDSGVFSHD VVRNGSLTGV
ALYGNHLYLT EQNTNTVWTC EVGSDGDPIA CHSHVALSAN CSIYGPIGIA ATQQGIFVVA
RGPAKQGTIC WFDLQGHKIA EVDGEYVDIT STRSGDLLAA TQNELHRVST DGNKLTTKRF
AGGSTNSCLP NTEGDDTLLC EITRLLVVTE YEMYVTSEKK SVLRSVTLPP VYVQGLFPGR
PLPVGYPDKD IMEWIVGNLT EDINTALGTT ESIVASSSVH VDSTTWLTNF TAGVQQPDFD
DEKTEQALHE SNYEHTKEAA DEYYNLTDEQ VYMDSTMVPY CNRLSLDALR RKLAKEAGEV
LNFTLIYADM PLKAESSDAE NITTVKLLMP ASFNNTVTHD LLSDANLTET AHSFIKYLRS
SDTHVDVTFS NPPFNFSSLT PDEEQEVRWY IHDEVMNQIK KCEERSTGRS MARREEVGDH
SRTTIATALD SNVTGVCQST ITNRTVSLFY QPPYVEMSLY EVFIPGNYTF DVSECVGEID
WQDLNDHLNN DTVRPTTEKA PKCGRVCLII IAVVCALIVA VLIVLAVVFT SKRRRLAAVV
APARPKFVST LDEDEQDYAS AYGNKERVEQ
```

B

```
Tb927.5.4580     -----MCFIFGVEMSNLAKRPMSLRKLPQLLLLIMIGIAFVAVECIGAPVKLPRRVDTVA
Tb927.8.4050     MPLWKQTNCEVETMNVREVVGTVHLGYVSQMLLLVATVATIVVRSGAAPIELKRHVTTVA
                              *.             . :**::  :* :.*.. .**::* *:* ***

Tb927.5.4580     GQFGFDGTTDGSSNVSMLSSPYALCRGRTNDEILVGSSNSFRNYSRKTKETGTFLRGGPT
Tb927.8.4050     GKYGHIGDKDGFPGMSELSSPHAMCRGRNSDEILLGTVDRFRAFSRKNRETTTITAWETD
                 *::*. * .** ..:* ****:*:****..****:*: : ** :***.:** *:    .

Tb927.5.4580     GG--LVSADAKISKPRSCVRRGSGNHTIIYFVDDQNGLKYINDNEIQHVTVGNGLSLTSV
Tb927.8.4050     EDQKSGSRSVKVDKPRACVQWTVGGSTFVYFVESMGEVKYFKDSGVFSHDVVRNGSLTGV
                  .    * ..*:.***:**:   *. *::***:. . :**::*. :    * .. ***.*

Tb927.5.4580     AIYEKDLYVTDQNNKSVWRCNVGGAGKPQNCEEKKFTGLTFTAK-PEGIAVTSKGIFVAA
Tb927.8.4050     ALYGNHLYLTEQNTNTVWTCEVGSDGDPIACHSHVALSANCSIYGPIGIAATQQGIFVVA
                 *:* :.**:*:**.::** *:**. *.*  *..:   . . :   * ***.*.:****.*

Tb927.5.4580     RDSSNKGALLWLDMNGGGSKGNVSGGFVDVFSTESGMLYAATEKELYTVTATGSAFSVTS
Tb927.8.4050     RGPAKQGTICWFDLQG-HKIAEVDGEYVDITSTRSGDLLAATQNELHRVSTDGNKLTTKR
                 *..:::*:: *:*::*  . .:*.* :**: **.** * ***::**: *:: *. ::..

Tb927.5.4580     FAGKNTSSCYSHANGEDIVLCDNSRLLVIEEYEMYVTSKEKHTMRALTLPPVNLTAIFRG
Tb927.8.4050     FAGGSTNSCLPNTEGDDTLLCEITRLLVVTEYEMYVTSEKKSVLRSVTLPPVYVQGLFPG
                 *** .*.** .:::*:* :**: :****: ********::* .:*::***** : .:* *

Tb927.5.4580     RPAPVGYPNTTIMEQFVASLTEDVNKALGTNDSYVDPDSVRVDPDTWETNFTVFVQQTRF
Tb927.8.4050     RPLPVGYPDKDIMEWIVGNLTEDINTALGTTESIVASSSVHVDSTTWLTNFTAGVQQPDF
                 ** *****:. *** :*..****:*.****.:* * ..**:**. ** ****. ***. *

Tb927.5.4580     DN-TTEEKLRSLTYTQTDKTVDEYYGLTDEYVYIDTVLVPFCDDASLVTIQRALAREAGR
Tb927.8.4050     DDEKTEQALHESNYEHTKEAADEYYNLTDEQVYMDSTMVPYCNRLSLDALRRKLAKEAGE
                 *: .**: *:. .* :*.::.****.**** **:*:.:**:*:  ** :::* **:***.

Tb927.5.4580     ALNFSLIYADKPITFGSDVAENVTAVKLLMPHSFKNATTPKQLSAANLTDFAHNLVKDLR
Tb927.8.4050     VLNFTLIYADMPLKAESSDAENITTVKLLMPASFNNTVTHDLLSDANLTETAHSFIKYLR
                 .***:***** *:.  *. ***:*:****** **:*:.* . ** ****: **.::* **

Tb927.5.4580     ASDTRVDITFPDPPFNFSAVVPEREQEVRWFVHGKVMKQLEICERLGSQGDAAVIAAAAA
Tb927.8.4050     SSDTHVDVTFSNPPFNFSSLTPDEEQEVRWYIHDEVMNQIKKCEERS-------------
                 :***:**:**.:******::.*:.******::*.:**:*:: **. .

Tb927.5.4580     ATARGKANVTLNTSGVKANDTGVGPNTTNTAGGANTTANVVANGTANVIVNPSTNATPTG
Tb927.8.4050     ----------------------------------------------------------TG
                                                                           **

Tb927.5.4580     TTNASVTNTTERAVPVVAPTQPSNGYAECRSAITNRTETQNMEPPYDRKHRYEVFLPKKY
Tb927.8.4050     RSMARREEVGDHSRTTIATALDSNVTGVCQSTITNRTVSLFYQPPYVEMSLYEVFIPGNY
                  : *   :. ::: ..:*.:  **  . *:*:***** :   :*** .   ****:* :*

Tb927.5.4580     DFNVSWCVDIIDWRDLDEMLNNRTDEVVEKSLSWCGHGCIIAFAVVGSLIAACLVVLAVV
Tb927.8.4050     TFDVSECVGEIDWQDLNDHLNNDTVRPTTEKAPKCGRVCLIIIAVVCALIVAVLIVLAVV
                  *:** **. ***:**:: *** * . . :. . **: *:* :*** :**.* *:*****

Tb927.5.4580     LTSKRRRLAAVVAPPRPKFVSTVEDDEEDRVSNIGVPLTDGKGTTAP
Tb927.8.4050     FTSKRRRLAAVVAPARPKFVSTLDEDEQDYASAYGNKERVEQ-----
                 :*************.*******:::**:* .*  *      :
```
