# Supplementary figures and images for: Identification and Characterization of a Stage Specific Membrane Protein Involved in Flagellar Attachment in Trypanosoma brucei
Source: PLoS One. 2013 Jan 15;8(1):e52846. doi: 10.1371/journal.pone.0052846 (PMC3546053; doi:10.1371/journal.pone.0052846)

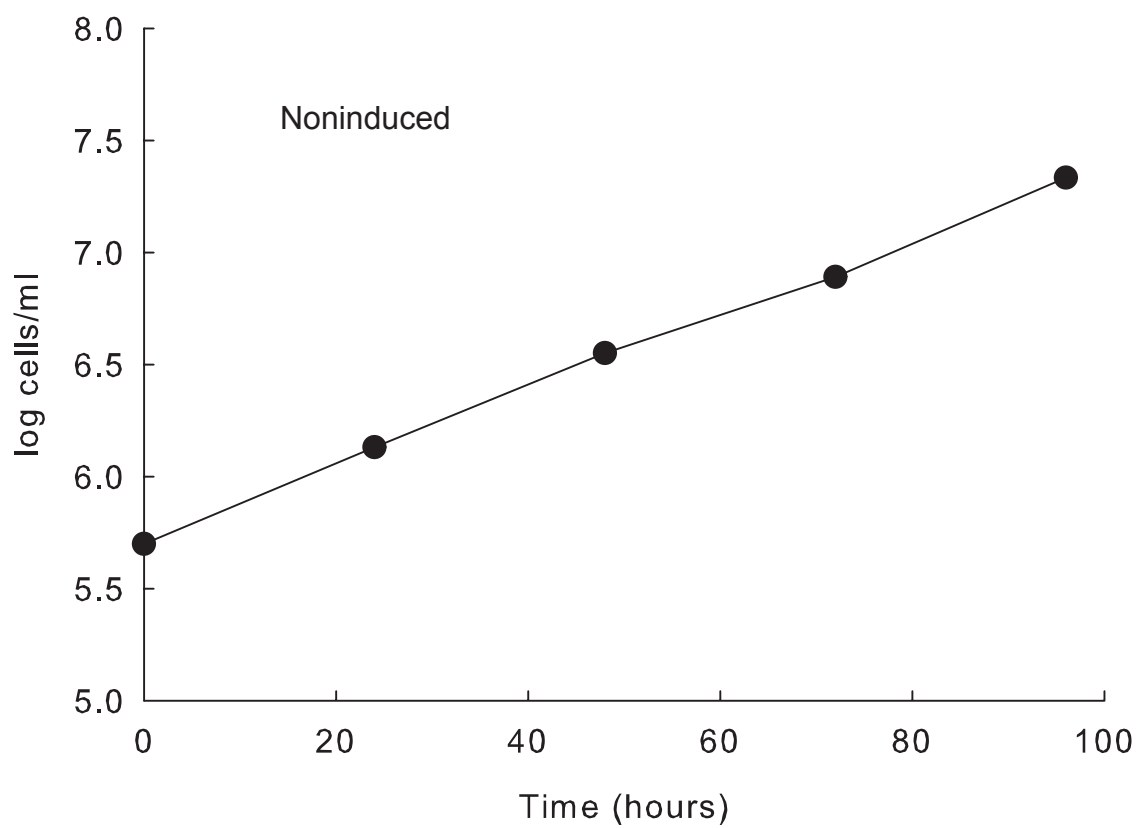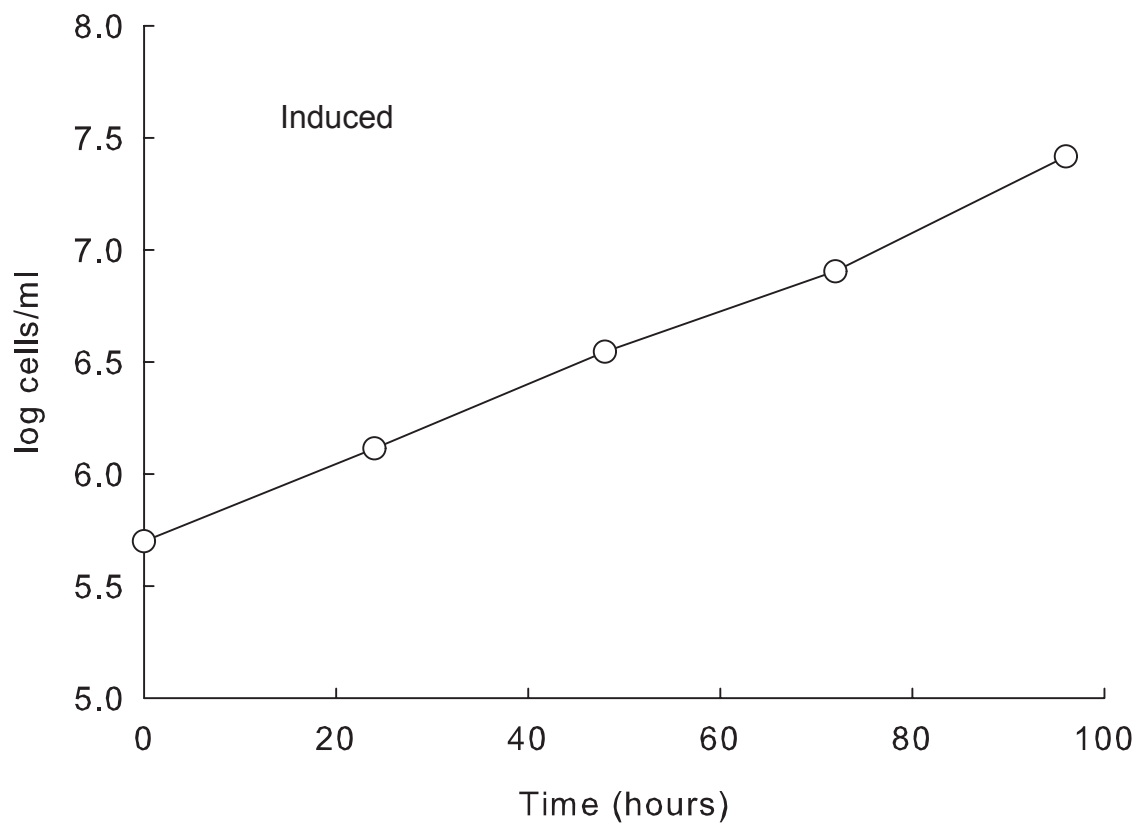

Supplement: Figure S4 — Knockdown of FLA3 does not affect the growth of procyclic forms of T. brucei. Knock down of residual FLA3 mRNA had no effect on the growth of a cloned FLA3 RNAi procyclic cell line cultured in the presence (○) or absence of tetracycline (•). A decrease in the residual FLA3 transcript level was confirmed by qRT-PCR which gave a relative ratio (induced/noninduced) of 0.53±0.23 (mean ± SD of four determinations). (PDF) [file pone.0052846.s004.pdf]

0 h

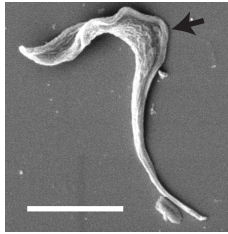

12 h

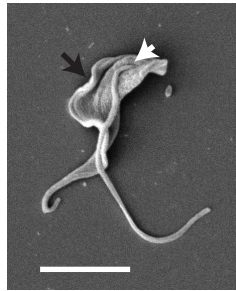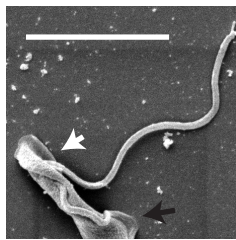

18 h

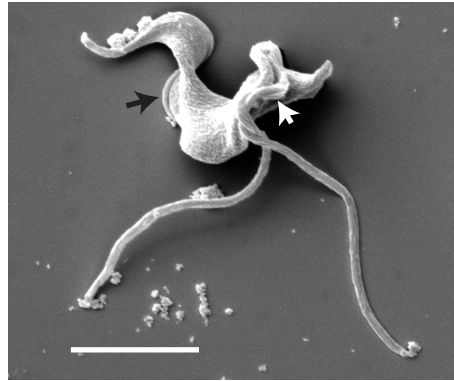

Supplement: Figure S5 — Analysis of flagellar detachment in FLA3 bloodstream RNAi cells using scanning electron microscopy. The cells were fixed at various times after the induction of the FLA3 dsRNA and processed for analysis by scanning electron microscopy. At the zero time the cells had the typical long slender morphology with the flagellum (black arrow) emerging from the flagellar pocket at the posterior end of the cell and remained attached along the entire cell body until it extended as a free flagellum at the anterior end of the cell. After 12 hours most of the cells had a detached flagellum (white arrow) but also retained an attached flagellum (black arrow). In many cases the new flagellum was fully detached and emerged from the cell posterior to the existing flagellum which remained attached to the cell body. At 18 hours post induction many of the cells possessed multiple detached flagella (white arrow) but still retained an attached flagellum (black arrow). The failure of cytokinesis was obvious at this stage by the abnormal morphology and large size of these cells. Bar = 10 µm. (PDF) [file pone.0052846.s005.pdf]

A

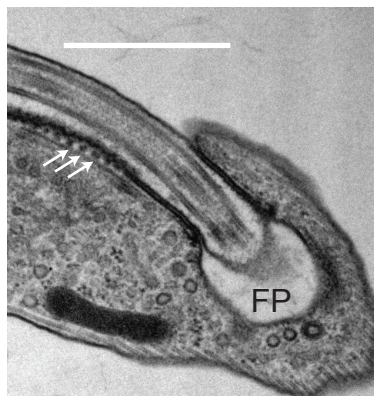

B

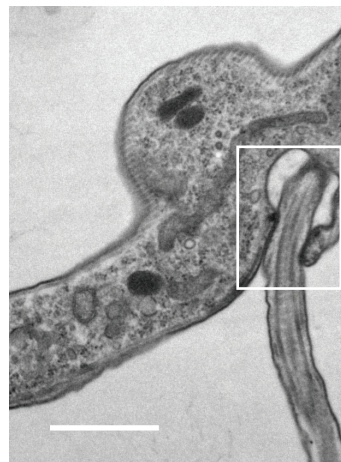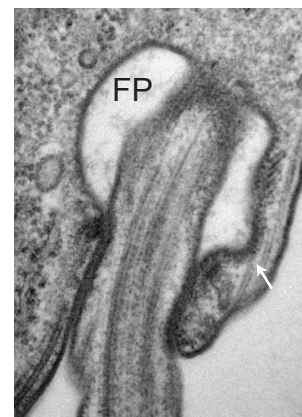

Supplement: Figure S6 — Flagellar pocket region of a detached flagellum. The cells were cultured for 24 h in the presence of tetracycline, fixed and processed for transmission electron microscopy. Panel A. Section through the flagellar pocket of a non induced FLA3 RNAi cell. The flagellum is tightly associated with the neck of the flagellar pocket (FP). After emerging from the pocket the flagellum is bend and attached to the cell surface. The regularly spaced structure of the FAZ filament is clearly visible along the attachment zone (arrows). Panel B. Section through the flagellar pocket of a completely detached flagellum. The flagellum remains tightly associated with the neck of the pocket (boxed region) but fails to attach to the surface and extents as a completely free flagellum. The flagellar pocket possesses has a normal morphology. The arrow indicates apparent formation of coated vesicles which is indicative of endocytic activity. The boxed region is shown on the left. Bar = 1µm. (PDF) [file pone.0052846.s006.pdf]
